# Genomic Alterations and Their Implications on Survival in Nonmetastatic Colorectal Cancer: Status Quo and Future Perspectives

**DOI:** 10.3390/cancers12082001

**Published:** 2020-07-22

**Authors:** Reetu Mukherji, John L. Marshall, Andreas Seeber

**Affiliations:** 1 Ruesch Center for The Cure of Gastrointestinal Cancers, Lombardi Comprehensive Cancer Center, Georgetown University Medical Center, Washington, DC 20057, USA; reetu.mukherji@gunet.georgetown.edu; 2 Department of Hematology and Oncology, Comprehensive Cancer Center Innsbruck, Medical University of Innsbruck, Innsbruck A-6020, Austria; andreas.seeber@tirol-kliniken.at

**Keywords:** colorectal cancer, adjuvant, chemotherapy, microsatellite instability, genomic, prognosis

## Abstract

The selection of treatment according to genomic alterations is a standard approach in metastatic colorectal cancer but is only starting to have an impact in the earlier stages of the disease. The status if genes like *KRAS, BRAF,* and MMR has substantial survival implications, and concerted research efforts have revolutionized treatment towards precision oncology. In contrast, a genomic-based approach has not changed the adjuvant setting after curative tumor-resection in the daily routine so far. This review focuses on the current knowledge regarding prognostic and predictive genomic biomarkers in patients with locally advanced nonmetastasized colorectal cancer. Furthermore, we provide an outlook on future challenges for a personalized adjuvant treatment approach in patients with colorectal cancer.

## 1. Introduction

Molecular profiling and precision medicine have become a critical new standard of care in the management of patients with metastatic colorectal cancer (CRC). As is the case in other solid tumors, several key molecular abnormalities provide both prognostic and predictive support for therapeutic interventions in the management of metastatic disease. Diagnostic biomarkers determine whether a patient has CRC, prognostic biomarkers predict the likely or expected outcomes of the disease irrespective of treatment, and predictive biomarkers provide information on specific treatment responses in the presence or absence of the specified biomarker. However, few of these biomarkers are routinely used in the management of patients with stage I–III CRC (see Table 1). Up until today, virtually all clinical trials were performed in an unenriched patient population using only pathologic stage as the determining inclusion criteria. Stratifications have not been included for significant genetic abnormalities, nor have we included the still mystical distinction of right-sided versus left-sided colon cancers in clinical trials. Therefore, we are forced to use retrospective analyses to guide today’s practice. With the exception of microsatellite instability (MSI), no specific clinical trials have been performed in the adjuvant setting focusing on particular molecular abnormalities. In this review, we will discuss current knowledge of key molecular markers and the role that they play either prognostically or predictively in the management of patients with non-metastatic CRC.

## 2. MSI/MMR Status

Mismatch repair (MMR) alterations have quickly become the most important new discovery in CRC biology and treatment. MMR is an important enzyme system responsible for maintaining genomic stability and correcting erroneous single-nucleotide mismatches during DNA replication that otherwise may result in somatic or germline mutations. Mutations in MMR genes impair the system’s ability to correct these errors during replication and thereby result in accelerated accumulation of mutations, especially in microsatellites. Microsatellites-numerous repeat-short sequences of nucleotide base-pairs scattered throughout the genome are susceptible to errors in replication and heavily rely on the MMR system. Germline or somatic mutations in MMR genes result in MMR deficiency (dMMR), which can lead to microsatellite hypermutation and length alterations, resulting in MSI. A tumor with an MSI-high (MSI-H) phenotype (instability in >30% of microsatellites examined) suggests dMMR. In contrast, an MSI-stable or -low (MSS or MSI-L) phenotype (instability in <30% of microsatellites) indicates proficient MMR (pMMR) [1]. Lynch syndrome, an inherited syndrome caused by autosomal dominantly inherited germline mutations in one of the MMR genes (*hMLH1, hMSH2, hMSH6, hPMS2, EPCAM),* is seen in 3% of all CRC patients, whereas sporadic MSI-H status, most commonly due to inactivation of *hMLH1,* is seen in about 5–20% of all CRCs [2,3,4,5,6].

MSI-H status is more frequent in stage II than stage III CRCs (22% vs. 12%). It confers a reduced likelihood of nodal or distant metastasis, which is likely attributable to the presence of immunogenic neoantigens from hypermutation and increased lymphocytic infiltration, contributing to improved immune-surveillance and prevention of micro-metastases [7,8,9]. Numerous retrospective studies and systematic reviews suggest MSI-H status is associated with a better prognosis, particularly in stage II CRC [10,11,12,13]. For instance, in the Quick and Simple and Reliable (QUASAR) study, dMMR status was associated with a reduced risk of recurrence after resection in stage II CRCs (hazard ratio (HR) 0.31) [12]. This finding is in line with data coming from a broad systematic review, in which Popat et al. showed that MSI was associated with better overall survival (OS) in locally advanced CRC (HR 0.67) [13]. Thus, MSI/dMMR status is considered an independent predictor of survival, with evidence suggesting that favorable prognostic effects are more significant in stage II compared to stage III CRCs [14,15,16,17] (see also Table 1, Table 2 and Table 3).

Large meta-analyses suggest that high-risk CRCs—based on nodal status—derive more significant benefit from adjuvant therapy than less invasive disease. Thus, patients with stage III CRC had a 5-year disease-free survival (DFS) of 63.6% and 49.0% when treated with and without adjuvant therapy, respectively, compared to 81.4% and 79.3% in patients with stage II CRC [18]. Furthermore, although recommendations for adjuvant treatment of patients with non-subtyped stage III CRCs are established based on prior studies, the role for adjuvant therapy in patients with MSI-H stage III disease is as unclear as for stage II CRCs [19,20,21,22].

Various studies have shown that patients with MSS/MSI-L/pMMR CRCs benefit from adjuvant 5-fluorouracil (5-FU)-based treatment regimens, whereas those with MSI-H/dMMR CRCs do not [10,23,24]. The reason for 5-FU resistance in MSI-H/dMMR tumors remains unclear. 5-FU has multiple proposed mechanisms of action, including enzyme inhibition in the DNA replication pathway leading to dinucleotide imbalances and DNA damage. Furthermore, 5-FU is a pyrimidine analog misincorporated into DNA/RNA, resulting in impaired genomic processing [25]. Preclinical studies suggest that an intact MMR system may be required to recognize 5-FU and facilitate apoptosis and cytotoxicity, and dMMR may lead to 5-FU resistance [26,27,28]. For instance, Arnold et al. demonstrated that dMMR *hMLH1* hypermethylated human CRC cell lines that underwent *hMLH1* promoter demethylation with 5-Aza-2′-deoxycytidine overcame 5-FU resistance in vitro [28].

Results from a retrospective study carried out by Ribic et al. indicated that adjuvant therapy improved outcomes in patients with stage II and III MSI-L/MSS CRCs but worsened outcomes in patients with stage II and III MSI-H CRCs [23]. Another study showed a survival advantage in patients with stage III MSI-L/MSS CRCs but not in those with stage II MSI-L/MSS CRCs or stage II/III MSI-H/dMMR CRCs [10]. In a later study, investigators reported improved DFS with 5-FU-based adjuvant therapy in pMMR stage II and III CRC patients but not in those with dMMR [24]. In fact, consistent with the findings by Ribic et al., adjuvant therapy was associated with reduced OS in patients with stage II dMMR CRC compared to surgery alone (HR 2.95). On the other hand, investigators of the QUASAR trial found no association between MMR status within stage II CRC and patient benefit derived from adjuvant chemotherapy [12]. The lack of benefit in using adjuvant 5-FU-based therapy in tumors with MSI is also seen in a large systemic review [13]. One prospective study led to findings consistent with those in the aforementioned retrospective studies with better outcomes from adjuvant fluorouracil-based chemotherapy only with pMMR stage II and III CRCs and no benefit in dMMR CRCs [67]. A retrospective subgroup analysis of the PETACC-3 trial led to the discovery that adjuvant chemotherapy (5-FU ± irinotecan) of patients with stage II MSI-H/dMMR CRCs led to improved recurrence-free survival (RFS) but not OS [14]. No improvement in either RFS or OS was apparent in patients with stage III CRCs.

Nonetheless, the majority of studies suggest that, although MSI-H/dMMR status is associated with favorable patient prognosis, it is not predictive for response to adjuvant therapy, with some data suggesting detrimental outcomes with adjuvant treatment, especially in stage II disease [23,24,73,74,78]. Thus, the current National Comprehensive Cancer Network (NCCN) and European Society of Medical Oncology (ESMO) guidelines recommend against routine adjuvant therapy for MSI-H stage II CRC. In patients with stage II MSS/pMMR CRCs, the decision for adjuvant treatment (5-FU/leucovorin or capecitabine) should be based on the presence of other clinical high-risk factors for recurrence (e.g., localized perforation, bowel obstruction, neuronal/vascular invasion, lymph node status, and margin status), anticipated morbidity of treatment, and patient preference [22,81]. The current NCCN and ESMO guidelines recommend that patients with stage III CRCs should receive adjuvant therapy, irrespective of MSI/MMR status [18,82].

Recent studies suggest that MSI/MMR status and other molecular markers may affect responses to different adjuvant chemotherapies and influence outcomes amongst those high-risk stage II and III CRCs (see Table 3). As previously discussed, preclinical models suggest MSI may be a negative predictive marker for response to 5-FU [28,83,84], and human clinical studies suggested a lack of benefit with 5-FU therapy in dMMR stage II and III CRCs compared to Pmmr [24,67]. However, some preclinical models suggest MSI may confer higher sensitivity to irinotecan, a camptothecin derivative that inhibits DNA topoisomerase I and causes double-strand DNA breaks, inhibition of DNA synthesis, and arrest of the cell cycle in the synthesis and gap 2 phases of interphase [85,86,87]. For example, Vilar et al. demonstrated that human CRC cell lines that were MSI-H due to *hMLH1* inactivation were four to nine times more sensitive to topoisomerase I inhibition compared to MSS cell lines [87]. dMMR CRCs tend to accumulate mutations in genes involved in double-strand DNA break repair, and it is suggested that they are chemosensitized by agents such as irinotecan due to their faulty repair system [87,88]. In the prospective CALGB 89803 study, high-risk patients with stage II and III CRCs were randomized to adjuvant 5-FU/leucovorin (5-FU/LV) with and without irinotecan [89]. MMR status was not predictive of OS in either arm. However, in the 5-FU/LV plus irinotecan arm, a significantly better 5-year DFS was observed in patients with MSI-H/dMMR tumors than in patients with MSS tumors (HR 0.76 vs. 0.59). This trend was not seen in patients treated with 5-FU/LV alone. Among all MSI-H/dMMR patients on study, adding irinotecan to 5-FU/LV seemed to improve DFS (HR 0.76 vs. 0.57), and although this finding was not statistically significant, no such trend was observed in MSS patients (HR 0.59 vs. 0.61). The prognostic effect seen with irinotecan in the CALBG 89803 study was not observed in the PETACC-3 trial [16,90]. Multivariable analysis revealed that MSI-H status was an independent prognostic variable independent of sex, T stage, and grade for RFS and OS.

According to current guidelines, irinotecan is not recommended in the adjuvant setting. Therefore, although the CALGB 89803 trial results are clinically inapplicable, they suggest that MSI-H/dMMR CRCs have a unique biology that may affect prognosis even with different chemotherapy agents. Oxaliplatin, a platinum compound that causes DNA adducts and intra-strand cross-links between DNA base pairs, inhibition of DNA replication, and ultimately apoptosis, is commonly used in combination with 5-FU in CRC. Platinum agents, such as oxaliplatin, cisplatin, and carboplatin, interfere with the pMMR system, cause DNA damage, impair DNA repair, and ultimately result in apoptosis. dMMR confers resistance to cisplatin and carboplatin as cells can proliferate despite DNA damage from the chemotherapy [91]. However, unlike cisplatin and carboplatin, dMMR CRC cells in vitro were sensitive to oxaliplatin, and this was attributed to a unique, bulky 1,2-diaminocyclohexane ligand of oxaliplatin that prevents MMR complex binding, more effectively inhibits DNA synthesis, and ultimately results in apoptosis [92]. Subgroup analyses in retrospective human studies show that the addition of oxaliplatin to 5-FU may improve outcomes compared to 5-FU alone in dMMR CRCs, particularly in stage III disease. However, other studies did not reproduce these results [79,93]. It is possible that oxaliplatin and other cytotoxic agents offset potential 5-FU resistance in dMMR tumors, although more highly populated studies are required to prove this hypothesis. Although the mechanisms behind oxaliplatin and 5-FU synergism remain unclear, and interactions between oxaliplatin and dihydropyrimidine dehydrogenase (DPD) activity were not directly investigated in this study, one group hypothesized that oxaliplatin might downregulate DPD and decrease 5-FU catabolism [94].

Myriad studies suggest that the prognostic value of MSI/MMR status can be stratified based on the presence or absence of additional molecular markers. In the PETACC-3 trial—although the study only included adjuvant chemotherapy-treated patients—exploratory statistical models showed that stage III tumors with additional detected biomarkers such as MSI-H and simultaneously retained SMAD4 expression had comparable survival to stage IIA disease [16]. The authors suggested that with further validation using large multivariable analyses, molecular profiles, including MSI/MMR status, might contribute to prognostic assessment in stage II and III CRCs, as well as potentially determine those stage III patients that could be spared adjuvant chemotherapy. Watanabe et al. studied the molecular features of 460 high-risk stage II and stage III CRCs from two clinical trials (Eastern Cooperative Oncology Group protocol E2284 and protocol E2288), in which patients received fluorouracil-based adjuvant chemotherapy [77]. The authors reported a statistically significant improvement in 5-year DFS in patients with stage III CRCs that were MSI-H compared to MSS (64% vs. 49%, *p* = 0.02). Five-year OS, however, was not different. Among patients with MSI-H CRCs, significant 5-year DFS (79% vs. 40%, *p* = 0.007) and OS (74% vs. 46%, *p* = 0.04) advantages were noted in patients with a gene mutation in the type II receptor for TGF-β1. These findings suggest that stage III MSI-H CRCs concurrently harboring TGF-β1-mutations predict likelihood of survival in patients treated with adjuvant 5-FU-based chemotherapy, although the mechanism of the survival advantage is unclear. In a posthoc analysis of the CKVO 90-11 trial, almost 400 patients with stage III CRC were assessed for MSI as well as tumor protein 53 (*TP53*) mutations in exons 4 to 8 (the majority of mutations were in the DNA binding domain) and Kirsten rat sarcoma viral oncogene homolog (*KRAS*) mutations in exons 1 and 2. Both TP53 mutations and MSI-H were associated with a prolonged DFS in patients with 5-FU-based adjuvant treatment; however, in the multivariate analysis adjusted for tumor grade, histology, number of tumor-positive nodes, and depth of tumor invasion, only *TP53* remained a statistically significant independent predictor of DFS [61].

Although there are promising data presented here concerning MSI status and its predictive value, findings are derived predominantly from retrospective analyses of a limited patient population. As such, further large prospective trials to study MSI/MMR status and combinations with other molecular markers, including but not limited to *KRAS* or *BRAF*, are required to understand their prognostic values better. While some in vitro studies suggest the efficacy of different chemotherapies vary depending on dMMR status, the clinical implications of these results have yet to be determined. Future prospective studies analyzing these biomarkers and patient outcomes following treatment with different chemotherapy regimens may help guide the choice of adjuvant therapy.

## 3. *KRAS* Mutation

*KRAS* is a proto-oncogene that transduces—through its GTPase activity—extracellular stimuli from the epidermal growth factor receptor (EGFR) in the nucleus, triggering cell growth, proliferation, differentiation, and survival [95,96]. The *KRAS* mutation is one of the most frequently mutated genes in CRC, found in up to 40–50% of cases [97]. Furthermore, similar to mutated *APC* and *TP53*, the *KRAS* mutation is a crucial step in CRC carcinogenesis [98]. Although the detection of mutations in *KRAS* in metastatic disease is essential before making therapeutic decisions [21,22], the analysis of these aberrations in patients with a nonmetastatic disease is not a standard of care, and they have no clear clinical role [52,99]. Only recently, Scott et al. used the National Cancer Database (NCDB) to analyze clinical features and outcomes of patients with *KRAS-*mutated nonmetastatic CRC [45]. In this retrospective study, KRAS mutations were more commonly found in tumors from African American patients and in right-sided primary tumors. Moreover, *KRAS-*mutated stage III tumors were associated with worse patient outcomes than *KRAS* wild-type (wt) tumors. This finding is in line with a later study showing that both codon 12 and 13 *KRAS* mutations are related to right-sided stage III colon tumors and inferior patient survival [46]. Of note, right-sided tumor location alone was attributed to shorter survival-after-recurrence and OS independent of the *KRAS* status [47]. A later study confirmed that codon 12 *KRAS* mutations were associated with an inferior OS; however, codon 13 *KRAS* mutations had no impact on survival [48]. Deng and colleagues showed that detection of *KRAS* alterations in tumors of patients not receiving adjuvant chemotherapy had a worse 3-year DFS than patients with wt tumors. On the other hand, other analyses could not show any prognostic implications of *KRAS* mutations in nonmetastatic CRC.

Neither the CALGB 89803 nor the PETACC-3 trials have demonstrated a prognostic effect of *KRAS* mutations in patients receiving adjuvant chemotherapy [7,51]. In an ancillary study of the PETACC-8 trial, *KRAS* mutations in exon 2 (codons 12 and 13) were examined in patients with stage III CRC receiving adjuvant chemotherapy [100]. In line with data derived from the PETACC-3 trial, mutations in exon 3 (codons 12 and 13) were not prognostic. However, *KRAS* exon 2 mutations were associated with shorter time to recurrence. In the prospective NCCTG N0147 trial, patients were enrolled before molecular testing for colon cancer was established as standard-of-care. However, tumors from more than 2700 stage III CRC patients were retrospectively tested for mutations in *KRAS* and *BRAF*, as well as MMR status, and their relationships to patient outcomes were assessed [50]. Patients with pMMR tumors and a concomitant *BRAF* or *KRAS* mutation had a shorter 5-year DFS compared to patients without these alterations. Consistent with these data, further studies showed that particularly patients with pMMR and *KRAS* mutations had reduced survival compared to patients with pMMR and *KRAS* wt or dMMR CRC [53,101].

In summary, the prognostic value of *KRAS* mutations in nonmetastatic CRC remains debatable because of conflicting results from several studies. The current literature does not support the routine testing for *KRAS* in stage I-III CRCs. Study designs (mainly retrospective or posthoc analyses), tumor heterogeneity, primary tumor location, assessment of MMR, as well as adjuvant therapies received, could have influenced the prognostic value of *KRAS* mutations. Thus, to get a clear idea of the prognostic value of *KRAS* mutations, prospective trials investigating *KRAS* mutations stratified according to MMR and primary tumor location in the nonmetastatic setting are necessary.

### 3.1. BRAF Gene Alteration

The serine/threonine-protein kinase *BRAF* acts as a downstream effector of EGFR/*KRAS* signaling and interacts with the MEK/ERK pathway [102]. Roughly 10% of all CRC tumors possess a *BRAF* mutation [103]. The most frequently reported alteration results in a valine-to-glutamic acid (V600E) that activates the MEK/ERK pathway [104]. *BRAF* mutated CRC correlates with dMMR/MSI-H, right-sided tumors, female sex, and poor outcome. Notably, *BRAF* mutations are mutually exclusive of *KRAS* mutations [54,55,105,106,107]. The negative prognostic effect of *BRAF* was seen in several trials. In the PETACC-3 study, *BRAF* mutations were predictive of poor patient OS, particularly in the subpopulation of pMMR tumors, but were not prognostic for RFS [7]. In line with these results, the PETACC-8 trial showed that both *BRAF* and *KRAS* mutations are related to an inferior outcome in patients with pMMR but not for dMMR/MSI-H [55]. Several other studies confirmed that *BRAF* is associated with a worse outcome, especially in the pMMR subpopulation of stage III CRC [52,53,54,56,57,58,59,60]. Now that we are routinely using therapeutics against *BRAF*-mutated CRC in the metastatic setting, it would be highly relevant to study the effect of these agents in the adjuvant setting [36].

In summary, *BRAF* mutations seem to predict a worse outcome in patients with resected stage III CRC, especially in the subgroup of patients with a proficient MMR system. As such, future trials should use *BRAF* and MMR as important stratification markers.

### 3.2. TP53 Mutation

The so-called “guardian of the genome” *TP53* is a fundamental regulator of the cell cycle, apoptosis, and DNA repair [108]. Loss of function mutations in *TP53* are frequently observed during tumorigenesis of several different cancer entities, particularly in CRC, where *TP53* is a fundamental step during the adenoma-carcinoma sequence [109,110]. The impact of *TP53* gene alterations on the prognosis of CRC patients in the adjuvant setting has been widely analyzed [52,111]. Several studies have shown that *TP53* protein expression and gene mutations were associated with a poor outcome [61,62,63,64,65]. However, other studies could not replicate these results and found no correlation between *TP53* alterations and survival [112,113,114]. The prospective ABCSG-90 trial analyzed the prognostic efficacy of *TP53* in stage III CRC. *TP53* gene alterations were significantly associated with a good prognosis in patients with N1 tumors receiving adjuvant 5-FU-based chemotherapy, whereas *TP53* mutations in patients with N2 tumors were not prognostic [65]. However, one may argue that a *TP53* mutation is less responsible for prognosis than the nodal status as nodal status is a robust predictive clinical marker. Some preclinical studies suggest p53 expression may be essential to 5-FU induced apoptosis and contribute to oxaliplatin-induced apoptosis, although other studies report conflicting results [115,116,117].

Varying study designs and analytical methods used may be the reason for these conflicting results in clinical studies. Although some studies used sequencing techniques, others used immunohistochemistry (IHC) to detect protein expression of p53. However, using p53 overexpression as an indicator of *TP53* mutational status is weak because loss and gain of function mutations may lead to a loss of protein expression [118]. Furthermore, diagnostic antibodies used for the detection of p53 by IHC have varying sensitivity. Thus, further studies should focus on sequencing techniques to detect *TP53* and to further elucidate its prognostic effect in nonmetastatic CRC.

### 3.3. PIK3CA Gene Alteration

*PIK3CA* mutations activate the PI3K/AKT/mTOR pathway that constitutively leads to tumor cell proliferation and survival, as well as multidrug resistance [119,120]. *PIK3CA* mutation prevalence is higher in right-sided, *KRAS-*mutated, and MSI CRC [121]. In a retrospective study, Jian and colleagues showed that simultaneous mutations in *PIK3CA* and *TP53* are associated with poor survival in patients with stage III CRC compared to patients with wild type tumors [66]. However, single mutations in the *PIK3CA* gene did not affect outcome [122]. In the metastatic setting, PIK3CA gene alterations were correlated with primary resistance to chemotherapy and anti-EGFR therapy [123,124]. Recently, *PIK3CA* mutations have gained attention because, in May 2019, the US FDA approved a new PI3K-targeted therapeutic agent, alpelisib. Combination alpelisib and fulvestrant treatment yielded an impressive prolongation of PFS compared to fulvestrant monotherapy in patients with hormone-receptor-positive breast cancer [125]. For CRC patients, a phase I/II trial is currently ongoing, which is evaluating the efficacy of alpelisib in combination with encorafenib and cetuximab in the metastatic setting (NCT01719380). However, in the adjuvant setting, no study is presently planned or ongoing. Thus, with the currently available data, the prognostic and predictive value of *PIK3CA* remains unclear. Further analyses should focus on *PIK3CA* gene alterations in patients with stage III CRC receiving adjuvant chemotherapy since primary resistance to chemotherapy was described in the metastatic setting.

### 3.4. APC Mutation

Gene alterations in adenomatous-polyposis-coli (*APC*) is one of the most commonly found mutations in CRC [126]. *APC* is a key negative regulator of WNT signaling that results in activation of β-catenin and c-myc [127]. Its prognostic and predictive value in CRC remains inconclusive so far. Small retrospective studies showed that *APC* mutations are neither associated with survival nor with response to treatment in CRC [70,71,72]. On the other hand, Jorissen and colleagues showed that mutations in *APC* in MSS tumors had a favorable outcome compared to *APC* wild-type MSS tumors [69]. In contrast to these results, a further large retrospective study using targeted parallel sequencing revealed that *APC* mutations are associated with a worse survival in patients with stage III CRC treated with 5-FU [68]. As such, although APC mutations are one of the most relevant gene alterations in CRC, the prognostic and predictive value remains unclear.

## 4. Perspectives and Conclusions

The heterogeneous molecular landscape of metastatic CRC has dramatically changed the treatment and prognosis of patients [21,22]. With the approval of anti-EGFR and anti-VEGF agents in the first-line setting, stratified by *RAS* and *RAF,* survival of more than 30 months can be achieved [128,129,130]. However, the use of genetic biomarkers for guiding treatment decisions in early-stage disease is not currently implemented because data about the prognostic and predictive value of *KRAS*, *BRAF*, *TP53, APC,* and *PIK3CA* remain inconsistent.

The MSI-H/dMMR status—contributing to a favorable immunogenic environment—is an established predictive biomarker for response to checkpoint inhibitor therapy in various cancers, including CRC [131]. Although improved responses with immunotherapy have been reported in patients with metastatic MSI-H/dMMR CRCs, these responses are not seen in the majority of MSS/pMMR CRCs. Numerous trials of combined immunotherapy with other agents aimed at “sensitizing” these tumors are ongoing [132,133].

Based on trials reporting improved outcomes with single or combination immunotherapy, these treatments are included in the NCCN guidelines as second-line therapy for patients with metastatic MSI-H/dMMR CRC. Furthermore, promising results from a recently presented phase III trial investigating the checkpoint inhibitor pembrolizumab in the first-line setting of patients with MSI-H/dMMR metastatic CRC have given rise to a new standard of care for this patient subset [30].

Immunotherapy’s role in nonmetastatic CRC remains under investigation. In the ongoing phase II NICHE trial, investigators are evaluating its role in the neoadjuvant setting with early positive results [134]. The ongoing phase III ATOMIC trial (NCT02912559) is focused on the study of chemotherapy (modified FOLFOX) with or without immunotherapy (atezolizumab) in the adjuvant setting for dMMR stage III CRC [135]. Whether adjuvant immunotherapy improves outcomes or MSI/MMR status plays a predictive role in this setting has yet to be determined, pending the results of the ATOMIC trial and ideally more prospective adjuvant trials. Furthermore, there is evidence that tumor-intrinsic oncogenic signaling pathways also play a role in regulating the tumor microenvironment and immune system evasion [136,137]. For example, upregulation and activation of the β-catenin, STAT3, and the PI3K/AKT pathways were found to alter tumor T-cell infiltration and contribute to immune evasion when studied in various cancer types [136,138,139,140,141]. A study also demonstrated that oncogenic KRAS signaling is associated with increased immune evasion by upregulating programmed cell death ligand 1 (PD-L1) expression [142]. Even the renin-angiotensin system is thought to contribute to an immunosuppressive microenvironment, and while combining angiotensin-converting enzyme inhibitors with immunotherapy is a proposed therapeutic intervention, preclinical and clinical studies are lacking to date [143]. Further elucidation of these pathways and biomarker identifications may allow us to manipulate tumor immunotherapy responsiveness with combined therapeutic and “sensitizing” agents in the metastatic and adjuvant settings in the future.

Taken together, although molecular profiling changed how patients with CRC are managed and treated in the metastatic setting, the role of profiling in the adjuvant setting remains unclear. However, several studies are ongoing, and investigators will try to deliver new insights into the prognostic and predictive efficacy of molecular biomarkers in adjuvant therapy.

## Figures and Tables

**Table 1:** Biomarker Testing Currently Recommended Based on CRC Stage.

Stage of CRC	Biomarkers Routinely Tested	Biomarker Test Utilized
I	MMR/MSI status	**MSI** [29,30]: PCR or validated NGS panel. Loss of protein expression with IHC testing of the four MMR genes (*MLH1, MSH2, MSH6, PMS2*) should guide further genetic testing Instability in > 30% of microsatellites examined with PCR indicates dMMR and instability in <30% of microsatellites indicates proficient MMR [1].
II	MMR/MSI status	
III	MMR/MSI status	
IV	MMR/MSI status	
	KRAS	**KRAS, NRAS, BRAF** [31,32,33,34,35,36]: No specific methodology recommended (e.g., sequencing, hybridization)
	NRAS	
	BRAF	
	HER2 amplifications	**HER2 amplification** [37,38,39]: IHC, FISH, or NGS; ICH positive defined as 3+ staining (intense membrane staining that can be circumferential, basolateral, or lateral) in more than 50% of tumor cells). Score of 2+ should be reflexed to FISH; FISH positive if HER2:CEP17 ratio ≥ 2 in more than 50% of cells
	NTRK fusion	**NTRK fusion** [40,41,42,43,44]: IHC, FISH, DNA-based NGS, RNA-based NGS

Glossary: PCR; polymerase chain reaction, MMR; mismatch repair, MSI; microsatellite instability, NGS; next-generation sequencing, IHC; immunohistochemistry, KRAS; Kirsten rat sarcoma viral oncogene homolog, HER2; human epidermal growth factor receptor 2, FISH; fluorescence in situ hybridization

**Table 2:** Prognostic and Predictive Molecular Biomarkers Analyzed in Nonmetastatic Colorectal Cance.

Biomarker	Prognostic Value	Predictive Value
KRAS	Poor outcome in terms of DFS and OS [45,46,47,48,49]; In pMMR and KRAS-mutated subgroup DFS shorter [50]	No predictive value for patients treated with adjuvant 5-FU [7,51]
BRAF	Poor outcome in terms of OS in pMMR tumors [7,52,53,54,55,56,57,58,59,60]	No predictive value
TP53	Poor outcome with shorter OS, DFS and RFS [61,62,63,64,65]	Positive predictive biomarker for N1 tumors receiving adjuvant 5-FU [65]
PIK3CA	Poor prognostic marker only with co-mutations in TP53 [66]	No predictive value
MSI-H/dMMR	Associated with a better outcome [8,10,11,12,13,30], especially in stage II CRC [14,15,16,17]	Conflicting results: Shorter survival in patients treated with adjuvant 5-FU [10,23,24,67] Stage II CRC had improved RFS treated with 5-FU + irinotecan [14]
APC	Conflicting results: Poor prognostic marker in 5-FU treated stage III CRC [68] Associated with better outcomes in MSS tumors [69] No prognostic value [70,71,72]	No predictive value

Glossary: DFS; disease-free survival, OS; overall survival, RFS; recurrence-free survival, pMMR; proficient mismatch repair, 5-FU; 5-fluorouracil, CRC; colorectal cancer

**Table 3:** Prognostic and Predictive Data Based on MSI Status in Stage II and III CRCs.

Reference Study Type Population (*n*)	Prognostic Data and Implications		Predictive Data and Implications	
	Stage II	Stage III	Stage II	Stage III
[10] Retrospective Total, *n* = 953 MSI-H, *n* = 208 MSS, *n* = 745	*MSI-H (n = 120)* vs. *MSS (n = 371)* 5-FU treated and no ACT; Cumulative survival Log rank 10.5, *p* = 0.001 **yes survival benefit with MSI-H**	*MSI-H (n = 88)* vs. *MSS (n = 374)* 5-FU treated and no ACT Cumulative survival Log rank 7.25, *p* = 0.007 **yes survival benefit with MSI-H**	*5-FU treated* vs. *no ACT* MSI-H 5-year survival Log rank 2.0, *p* = 0.151 **no survival benefit, trend for worse outcome with 5-FU** MSS 5-year survival Log rank 0.97, *p* = 0.323 **no survival benefit with 5-FU**	*5-FU treated* vs. *no ACT* MSI-H 5-year survival Not reported, but per discussion no statistically significant difference **no significant survival benefit with 5-FU** MSS 5-year survival Log rank 5.39, *p* = 0.02 **yes survival benefit with 5-FU**
[23] Retrospective Total, *n* = 570 MSI-H, *n* = 95MSS/MSI-L, *n* = 475	*MSI-H* vs. *MSS* 5-FU treated and no ACT HR for OS 0.61 (95% CI 0.38–0.96) for combined stage II and III CRC (no by-stage analysis reported) No ACT HR for OS 0.32 (95% CI 0.14–0.75) for combined stage II and III CRC (no by-stage analysis reported)	*MSI-H* vs. *MSS* 5-FU treated and no ACT HR for OS 0.61 (95% CI 0.38–0.96) for combined stage II and III CRC (no by-stage analysis reported) No ACT HR for OS 0.32 (95% CI 0.14–0.75) for combined stage II and III CRC (no by-stage analysis reported)	*5-FU treated* vs. *no ACT* MSI-H HR for death 3.28 (95% CI 0.86–12.48) **no survival benefit, but trend for worse outcome with 5-FU** MSS/MSI-L HR for death 0.67 (95% CI 0.39–1.15) **no survival benefit, but trend for improved outcome with 5-FU**	*5-FU treated* vs. *no ACT* MSI-H HR for death 1.42 (95% CI 0.36–5.56) **no survival benefit, but trend for worse outcome with 5-FU** MSS/MSI-L HR for death 0.69 (95% CI 0.47–1.01) **no survival benefit, but trend for improved outcome with 5-FU**
[24] Prospective analysis of data on patients previously enrolled in 5 completed randomized clinical trials Total, *n* = 457 dMMR, *n* = 70 pMMR, *n* = 387 Analysis of data pooled with prior data from four of the five clinical trials [23]	*MSI-H* vs. *MSS* No by-stage analysis reported	*MSI-H* vs. *MSS* No by-stage analysis reported	*5-FU treated* vs. *no ACT* *Prospective analysis data* dMMR inadequate sample size prohibited analysis pMMR HR for 5-year DFS 1.01 (95% CI 0.56–1.83) **no survival benefit with 5-FU**	*5-FU treated* vs. *no ACT* *Prospective analysis data* dMMR inadequate sample size prohibited analysis pMMR HR for 5-year DFS 0.56 (95% CI 0.37–0.83) HR for OS consistent with DFS **yes survival benefit with 5-FU**
Pooled data: Total, *n* = 1027 dMMR, *n* = 165 pMMR*, n* = 862			*5-FU treated* vs. *no ACT* *Pooled data* dMMR HR for 5-year DFS 2.30 (95% CI 0.84–6.24) HR for OS 2.95 (95% CI 1.02–8.54) **no survival benefit, but trend for worse DFS and statistically significant decreased OS with 5-FU** pMMR HR for 5-year DFS 0.84 (95% CI 0.57–1.24) HR for OS reportedly consistent with DFS findings **no survival benefit with 5-FU**	*5-FU treated* vs. *no ACT* *Pooled data* dMMR HR for 5-year DFS 1.01 (95% CI 0.41–2.51) HR for OS reportedly consistent with DFS findings **no survival benefit with 5-FU** pMMR HR for 5-year DFS 0.64 (95% CI 0.48–0.84) HR for OS reportedly consistent with DFS findings **yes survival benefit with 5-FU**
[67] Prospective Total, *n* = 505 dMMR, *n* = 53 pMMR, *n* = 452 (9 out of 505 patients excluded from benefit of ACT analysis as they did not get 5-FU)	*dMMR (n = 35)* vs. *pMMR (n = 261)* 5-FU treated and no ACT 80% vs. 75% DFS respectively, *p* = 0.5 89% vs. 85% OS respectively, *p* = 0.6 **no survival benefit, according to MMR status**	*dMMR (n = 18)* vs. *pMMR (n = 191)* 5-FU treated and no ACT 73% vs. 61% DFS respectively, *p* = 0.4 78% vs. 75% OS respectively, *p* = 0.8 **no survival benefit according to MMR status**	5*-FU treated* vs. *no ACT* dMMR no improvement in probability of survival (89.5% vs. 82.4%, *p* = 0.4) and DFS (73.7% vs. 79.4%, *p* = 0.9) with adjuvant therapy in stage II/III CRC Trend reportedly maintained in subgroup analysis stage II disease **no survival benefit with 5-FU**	*5-FU treated* vs. *no ACT* dMMR no improvement in probability of survival (89.5% vs. 82.4%, *p* = 0.4) and DFS (73.7% vs. 79.4%, *p* = 0.9) with adjuvant therapy in stage II/III CRC Trend reportedly maintained in subgroup analysis stage III disease **no survival benefit with 5-FU**
			pMMR improved probability of overall survival (87.1% vs. 73.5%, *p* = 0.0001) and DFS (73.9% vs. 64.0% *p* = 0.0004) with adjuvant therapy in stage II/III CRCs Trend reportedly maintained in subgroup analysis stage II disease **yes survival benefit with 5-FU**	pMMR improved probability of overall survival (87.1% vs. 73.5%, *p* = 0.0001) and DFS (73.9% vs. 64.0% *p* = 0.0004) with adjuvant therapy in stage II/III CRCs Trend reportedly maintained in subgroup analysis stage III disease **yes survival benefit with 5-FU**
[73] Retrospective based QUSAR subgroup analysis Total, *n* = 1913 dMMR*, n* = 218 pMMR, *n* = 1695	*dMMR (n = 167)* vs. *pMMR (n = 469) in right-sided CRC* 5-FU treated and no ACT Risk of recurrence ratio 0.44 (95% CI 0.29–0.67) **yes survival benefit with dMMR** 5-FU OR for recurrence 0.36 (95% CI 0.20–0.65) **yes survival benefit if dMMR**	not reported	*5-FU (n = 311)* vs. *no ACT (n = 325)* dMMR (*n* = 167) 2 year OR for recurrence 0.45 (95% CI 0.09–2.23) **no survival benefit, but trend for improved outcome with 5-FU** pMMR (*n* = 469) 2 year OR for recurrence 1.07 (95% CI 0.61–1.87) **no survival benefit with 5-FU**	Not reported
	No ACT OR for recurrence 0.54 (95% CI 0.30–0.97) **yes survival benefit if dMMR**			
[74] Retrospective cohort study for stage II CRC MSI, *n* = 843 MSS, *n* = 6121	*MSI* vs. *MSS* ACT (defined as any chemotherapy) and untreated HR for OS 0.69 (95% CI 0.54–0.89) **yes survival benefit if MSI**	No stage III data	*ACT (defined as any chemotherapy)* vs. *no ACT* MSI (*n* = 843) HR 0.85 (95% CI 0.39–1.83) **no survival benefit with ACT** MSS (*n* = 6162) HR 0.47 (95% 0.37–0.60) **yes survival benefit with ACT** MSS subset with no high-risk features (*n* = 3400) HR 0.44 (95% CI 0.28–0.71) **yes survival benefit with ACT**	No stage III data
[75] Retrospective Stage III only all treated with FOLFOX Total, *n* = 598 MSI-H, *n* = 50 MSS/MSI-L, *n* = 548	Not analyzed	*MSI-H (n = 50)* vs. *MSS/MSI-L (n = 548) treated with FOLFOX* HR for DFS 0.598 (95% CI 0.263–1.58) HR for OS 0.872 (95% CI 0.352–2.163)	Not analyzed	Not analyzed
		**no survival benefit, with trend for improved outcomes if MSI-H**		
[76] Retrospective Subgroup analysis Stage II and III ACT in this study was 5-FU or capecitabine monotherapy ± oxaliplatin Total, *n* = 654 MSI, *n* = 63 MSS, *n* = 591	Not analyzed	*MSI* vs. *MSS* ACT (*n* = 203) HR for RFS (recurrence) 2.60 (95% CI 1.27–5.35) HR for disease-specific survival DSS 2.35 (95% CI 0.68–8.09) **no survival benefit and worse RFS in ACT treated if MSI-H** No treatment (*n* = 96) HR for RFS 3.21 (95% CI 0.68–15.04) HR for DSS 0.75 (95% CI 0.09–5.77) **no survival benefit if no ACT and MSI-H**	Not analyzed	*ACT* vs. *no ACT* MSI HR for survival benefit 0.67 (95% CI 0.08–8.15) **no survival benefit, but trend for improved outcome with ACT** MSS HR for survival benefit 0.35 (95% CI 0.17–0.69) **yes survival benefit with ACT**
[77] Retrospective data from patients with 5-FU based ACT previously enrolled in 2 randomized trials (ECOG E2284 and E2288-adjuvant stage II/III studies with combinations of 5-FU with leucovorin or levamisole) Total (Stage III), *n* = 229 MSI-H (stage III), *n* = 73 MSI-L (stage III), *n* = 156	Not reported	*MSI (n = 73)* vs. *MSS (n = 156) (having received 5-FU based chemotherapy)* 64% and 49% 5-year DFS respectively (*p* = 0.02) 68% and 56% 5-year OS respectively (*p* = 0.20) **yes DFS benefit, with trend for improved OS if MSI**	Study reports no predictive value seen, possibly due to small sample size (*n* = 121) **no survival benefit between different 5-FU based regimens**	Study reports no relationship between survival after treatment with a particular regimen and presence of molecular markers (no data reported) **no survival benefit between different 5-FU based regimens**
[14] Retrospective subgroup analysis from PETACC 3-EORTC 40993-SAKK 60/00 trial Total, *n* = 1254 MSI-H, *n* = 190 MSS/MSI-L, *n* = 1064	*MSI-H* vs. *MSS/MSI-L* 5-FU ± irinotecan HR for RFS 0.26 (95% CI 0.11–0.65) HR for OS 0.153 (95% CI 0.037–0.631) **yes survival benefit with MSI-H** 5-FU HR for RFS 0.22 (95% CI 0.05–0.94) HR OS 0.17 (95% CI 0.02–1.26) **yes RFS survival benefit with 5-FU if MSI-H**	*MSI-H* vs. *MSS/MSI-L* 5-FU ± irinotecan HR for RFS 0.69 (95% CI 0.47–1.01) HR for OS 0.674 (95% CI 0.426–1.07) **no survival benefit with MSI-H, but trend for improved survival with MSI-H** 5-FU HR for RFS 0.59 (95% CI 0.34–1.02) HR for OS 0.54 (95% CI 0.28–1.07)	*5-FU/Irinotecan* vs. *5-FU* MSI-H HR for RFS 1.68 (95% CI 0.28–10.05) HR for OS 1.14 (95% CI 0.07–18.2) **no survival benefit with addition of irinotecan to 5-FU** MSS/MSI-LHR for RFS 1.25 (95% CI 0.76–2.06) HR for OS 1.30 (95% CI 0.71–2.39) **no survival benefit with addition of irinotecan to 5-FU**	*5-FU/Irinotecan* vs. *5-FU* MSI-H HR for RFS 1.17 (95% CI 0.56–2.43) HR for OS 1.34 (95% CI 0.56–3.24) **no survival benefit with addition of irinotecan to 5-FU** MSS/MSI-L HR for RFS 0.85 (95% CI 0.68–1.08) HR for OS 0.88 (95% CI 0.67–1.15) **no survival benefit with addition of irinotecan to 5-FU**
	5-FU/Irinotecan HR for RFS 0.29 (95% CI 0.09–0.96) HR OS 0.14 (95% CI 0.02–1.03) **yes RFS survival benefit with 5-FU/irinotecan if MSI-H**	**-no survival benefit but trend for improved RFS and OS with 5-FU if MSI-H** 5-FU/Irinotecan HR for RFS 0.82 (95% CI 0.48–1.40) HR for OS 0.85 (95% CI 0.45–1.58) **no survival benefit with 5-FU/irinotecan if MSI-H**		
[15] Retrospective Results of PETACC-3 Total, *n* = 1254 MSI-H, *n* = 190 MSS/MSI-L, *n* = 1064	*MSI-H* vs. *MSS/MSI-L* 5-FU ± irinotecan HR for RFS 0.26 (95% CI 0.10–0.65) HR for OS 0.16 (95% CI 0.04–0.64) **yes survival benefit if MSI-H** 5-FU HR for RFS 0.22 (95% CI 0.05–0.91) HR for OS 0.18 (95% 0.02–1.32)	*MSI-H* vs. *MSS/MSI-L* 5-FU ± irinotecan HR for RFS 0.67 (95% CI 0.46–0.99) HR for OS 0.70 (95% CI 0.44–1.09) **yes RFS survival benefit, with trend for OS benefit if MSI-H.** 5-FU HR for RFS 0.56 (95% CI 0.32–0.96) HR for OS 0.51 (0.26–1.00)	*5-FU* vs. *F-FU/Irinotecan* Study reports similar RFS and OS when stratified by MSI status; by stage analysis not reported	*5-FU* vs. *F-FU/Irinotecan* Study reports similar RFS and OS when stratified by MSI status; by stage analysis not reported
	**yes RFS survival benefit, with trend for OS benefit if MSI-H** 5-FU/Irinotecan HR for RFS 0.30 (95% CI 0.09–0.96) HR for OS 0.14 (95% CI 0.02–0.03) **yes RFS survival benefit, with trend for OS benefit if MSI-H** **prognosis for MSI remains regardless of treatment with 5-FU or F-FU/irinotecan**	**yes RFS survival benefit, with trend for OS benefit if MSI-H** 5-FU/Irinotecan HR for RFS 0.82 (95% CI 0.48–1.40) HR for OS 0.94 (95% CI 0.52–1.72) **no survival benefit, with slight trend for improved RFS if MSI-H** **not as significant of a survival benefit compared to MSI-H status in stage II**		
[7] Retrospective Subgroup analysis PETACC-3 Total, *n* = 1254 MSI-H, *n* = 190 MSS/MSI-L, *n* = 1064	*MSI-H (n = 86)* vs. *MSS/MSI-L (n = 309)* HR for RFS 0.27 (95% CI 0.10–0.72) HR for OS 0.14 (95% CI 0.03–0.64) **yes survival benefit if MSI-H**	*MSI-H (n = 104)* vs. *MSS/MSI-L (n = 755)* HR for RFS 0.59 (95% CI 0.38–0.91) HR for OS 0.48 (95% CI 0.28–0.81) **yes survival benefit if MSI-H**	*5-FU* vs. *5-FU/irinotecan* MSI-H Stratified data not included MSS/MSI-L HR for RFS 0.79 (95% CI 0.47–1.31) HR for OS 0.75 (95% CI 0.40–1.40) **no survival benefit with addition of irinotecan to 5-FU**	*5-FU* vs. *5-FU/irinotecan* MSI-H Stratified data not included MSS/MSI-L HR for RFS 1.19 (95% CI 0.94–1.51) HR for OS 1.17 (95% CI 0.88–1.55) **no survival benefit with addition of irinotecan to 5-FU**
[78] Prospective updated analysis on CALGB 9581 and CALGB 89803 Total, *n* = 3002 dMMR, *n* = 330 pMMR, *n* = 1515 CALGB 9581 (stage II) Total, *n* = 935 dMMR, *n* = 199 pMMR, *n* = 736 CALGB 89803 (stage III) Total, *n* = 910 dMMR, *n* = 131 pMMR, *n* = 779	*dMMR (n = 199)* vs. *pMMR (n = 736)* HR for 5y DFS 0.65 (95% CI 0.47–0.89) HR for 5y OS 0.76 (95%CI 0.54–1.07) **yes DFS benefit with dMMR with trend for improved OS**	*dMMR (n = 131)* vs. *pMMR(n = 779)* HR for 5y DFS 0.82 (95% CI 0.60–1.11) HR for 5y OS 0.88 (95% CI 0.63–1.22) **no survival benefit with dMMR**	*No ACT* vs. *MoAb 17-1A (clinical trial monoclonal antibody)* dMMR (*n* = 199) HR for 5y DFS 1.21 (95% CI 0.68–2.18) HR for 5y OS 1.22 (95% CI 0.66–2.26) **no survival benefit in untreated** pMMR (*n* = 736) HR for 5y DFS 0.96 (95% CI 0.74–1.24) HR for 5 y OS 1.03 (95% CI 0.78–1.37) **no survival benefit in untreated**	*5-FU/LV* vs. *IFL* dMMR (*n* = 131) HR for 5y DFS 0.68 (95% CI 0.38–1.20) HR for 5y OS 0.85 (95% CI 0.46–1.59) **no survival benefit, but slight trend in improved outcome with addition of irinotecan** pMMR (*n* = 779) HR for 5y DFS 1.09 (95% CI 0.88–1.34) HR for 5y OS 1.03 (95% CI 0.82–1.31) **no survival benefit with addition of irinotecan**
[79] Retrospective Subgroup analysis Stage II dMMR, *n* = 149 Stage III dMMR, *n* = 187	Not analyzed	Not analyzed	*5-FP or 5-FP + oxaliplatin* vs. *no ACT* dMMR (*n* = 149) HR for DFS 0.85 (95% CI 0.19–3.88) with 5-FP HR for DFS 0.13 (95% CI 0.02–1.05) with 5-FP+ oxaliplatin **no survival benefit with 5-FP or 5-FP+oxaliplatin, with trend for improved survival with oxaliplatin added to 5-FP**	*5-FP or 5-FP + oxaliplatin* vs. *no ACT* dMMR (*n* = 187) HR for DFS 0.66 (95% CI 0.29–1.50) with 5-FP HR for DFS 0.41 (95% CI 0.19–0.87) with 5-FP + oxaliplatin **yes survival benefit with 5-FP in combination with oxaliplatin but not 5-FP alone**
[80] Retrospective Subgroup analysis Stage III CRC only Total, *n* = 233 MSI, *n* = 32 MSS, *n* = 201	Not included	*MSI* vs. *MSS (5-FU ± oxaliplatin)* HR for DFS 1.09 (95% CI 0.48–2.13) **no survival benefit if MSI-H**	Not included	*FOLFOX (n = 109)* vs. *5-FU/LV (n = 124)* MSI HR for DFS 0.17 (95% CI 0.04–0.68) **yes survival benefit if oxaliplatin is added** MSS HR for DFS 0.66 (95% CI 0.38–1.15) **no survival benefit, but trend for improved DFS if oxaliplatin added**

Glossary: ACT; adjuvant chemotherapy, 5-FU; 5-fluorouracil, 5-FP; 5-fluoropyrimidine, LV; leucovorin, IFL; Irinotecan, 5-fluorouracil, leucovorin, CALGB; Cancer and Leukemia Group B, ECOG; Eastern Cooperative Oncology Group, OS; overall survival, DFS; disease-free survival; The underlined terms refer to a subgroup, the italicized terms indicate which independent factors are being assessed in that subgroup for prognostic/predictive value, the regular text details the survival data, and the bolded text is the data interpretation and summary.
